# Site-Specific Conjugation of Native Antibody: Transglutaminase-Mediated Modification of a Conserved Glutamine While Maintaining the Primary Sequence and Core Fc Glycan via Trimming with an Endoglycosidase

**DOI:** 10.1021/acs.bioconjchem.4c00013

**Published:** 2024-03-18

**Authors:** Amissi Sadiki, Shanshan Liu, Shefali R. Vaidya, Eric M. Kercher, Ryan T. Lang, James McIsaac, Bryan Q. Spring, Jared R. Auclair, Zhaohui Sunny Zhou

**Affiliations:** † Department of Chemistry and Chemical Biology, Barnett Institute of Chemical and Biological Analysis, Northeastern University, Boston, Massachusetts 02115, United States; ‡ Translational Biophotonics Cluster, Department of Physics, Department of Bioengineering, Northeastern University, Boston, Massachusetts 02115, United States

## Abstract

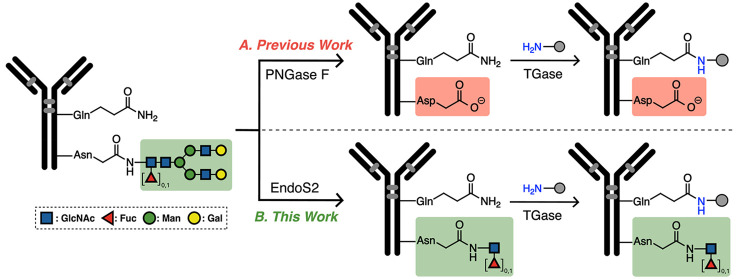

A versatile chemo-enzymatic tool to site-specifically modify native (nonengineered) antibodies is using transglutaminase (TGase, E.C. 2.3.2.13). With various amines as cosubstrates, this enzyme converts the unsubstituted side chain amide of glutamine (Gln or Q) in peptides and proteins into substituted amides (i.e., conjugates). A pleasant surprise is that only a single conserved glutamine (Gln295) in the Fc region of IgG is modified by microbial TGase (mTGase, EC 2.3.2.13), thereby providing a highly specific and generally applicable conjugation method. However, prior to the transamidation (access to the glutamine residue by mTGase), the steric hindrance from the nearby conserved N-glycan (Asn297 in IgG1) must be reduced. In previous approaches, amidase (PNGase F, EC 3.5.1.52) was used to completely remove the N-glycan. However, PNGase F also converts a net neutral asparagine (Asn297) to a negatively charged aspartic acid (Asp297). This charge alteration may markedly change the structure, function, and immunogenicity of an IgG antibody. In contrast, in our new method presented herein, the N-glycan is trimmed by an endoglycosidase (EndoS2, EC 3.2.1.96), hence retaining both the core N-acetylglucosamine (GlcNAc) moiety and the neutral asparaginyl amide. The trimmed glycan also reduces or abolishes Fc receptor-mediated functions, which results in better imaging agents by decreasing nonspecific binding to other cells (e.g., immune cells). Moreover, the remaining core glycan allows further derivatization such as glycan remodeling and dual conjugation. Practical and robust, our method generates conjugates in near quantitative yields, and both enzymes are commercially available.

As recently reviewed,^1−4^ both site-specific and nonspecific methods have been used to construct antibody conjugates for a plethora of applications; however, the former is gaining prominence for good reasons. Nonspecific methods—which include acylation of amines (e.g., lysines and the N-terminus) or alkylation of thiols (e.g., cysteines)—have been used to assemble various constructs, including approved antibody drug conjugates (ADCs).^1−3^ These approaches have several limitations. First, the reactions lack site-specificity and result in heterogeneous mixtures.^5^ Second, modification at or near the functional domains, such as the complementarity-determining regions (CDR) of antibodies, is likely to perturb the structure and thus function. These multiple sites of modification have been shown by our group and others^6−9^ to affect the protein’s activity negatively, for example, by reducing the binding affinity and specificity of antibody.^10^ Third, generally kinetically controlled, these processes also suffer from poor reproducibility and are prone to side-reactions.^5,11^ Fourth and last, characterization of nonspecific conjugation is cumbersome, as before conjugation, many proteins exhibit various post-translation modifications (PTMs) such as deamidation, oxidation, glycosylation or other reactive metabolites.^11−20^ Accounting for these PTMs, the number of unmodified and modified species (i.e., proteoforms) increases exponentially after nonspecific bioconjugation. As such, nonspecific conjugations make it difficult to detect all modifications and often leads to underestimation. For example, sites of modification are missed because of the prevalence of false negatives during analysis.^11^ To overcome these limitations, site-specific methods are needed.

Few methods exist to site-specifically conjugate native antibodies, especially without genetic engineering.^3^ In this context, native antibodies are defined as constructs that are either recombinantly produced or naturally existing in various species, whereby their primary amino acid sequence is conserved. Examples of such methods include Fc affinity peptide reagents to modify lysine^21,22^ and even fewer enzymatic approaches exist such as glycan remodelling.^3^ One such technique is using microbial transglutaminase (mTGase, EC 2.3.2.13, Uniprot P81453) to modify glutaminyl amide residues on antibodies. This enzyme catalyzes the formation of a substituted amide bond between an unsubstituted side-chain amide of glutamine residue (as an acyl donor) and various nucleophilic amine substrates (as an acyl acceptor).^23−27^

Despite possessing over 60 glutamine residues, native antibodies are poor substrates for mTGase, i.e., not modified. As such, it was a pleasant surprise that only a single glutamine (Q295 in IgG1) in the conserved Fc region of IgG is modified by mTGase, thereby providing a highly specific and generally applicable conjugation method.^28−31^ However, prior to the transamidation (access to the glutamine residue by mTGase), one has to reduce the steric hindrance from the nearby conserved N-glycan (N297 in IgG1; see Schemes 1 and 2). In previous reports, an amidase (PNGase F, EC 3.5.1.52) was used to completely remove the N-glycan.^31^ Of note, PNGase F also converts a net neutral asparagine (N297) to a negatively charged aspartic acid (D297 in IgG1; see Scheme 1). This charge alteration may markedly change the structure, function, and immunogenicity of the IgG antibody.

**Scheme 1:**
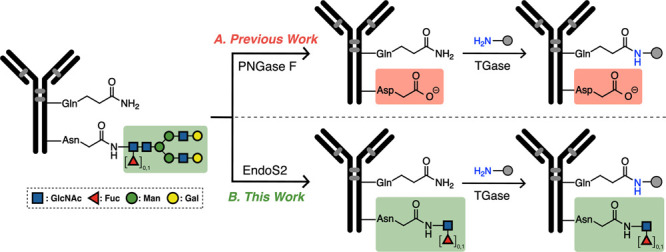
Two-Step Process In the two-step process, the initial step is to reduce the steric hindrance from the N-Glycan. In the previous work (A), the N-glycan is completely removed, and a neutral amide (Asn297) is converted to a negatively charged carboxylate (Asp297; highlighted in red) by an amidase (PNGase F). In our new method (B, this work), the primary sequence and core N-acetylglucosamine (GlcNAc) are preserved by an endoglycosidase (EndoS2). In the second step for both approaches, mTGase modifies antibody’s glutamine (Q295).

**Scheme 2:**
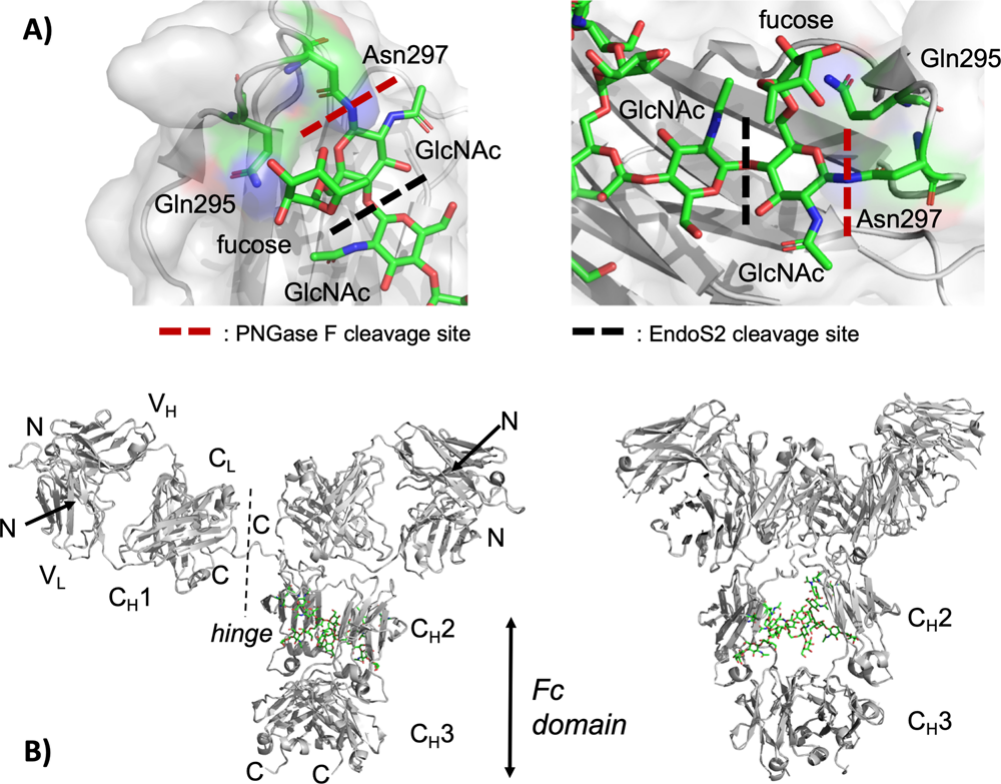
Structures of the Antibody around Glutamine Residue (Q295) Being Modified and the Nearby N-Glycan (A) Crystal structure of human antibody Fc fragment (PDB 4CDH(49)) highlighting the PNGase F and EndoS2-mediated cleavage sites on asparagine (N297) at the side chain amide bond and the innermost N- acetylglucosamine (GlcNAc), respectively. (B) Zoomed-out crystal structure of human antibody (PDB 1HZH(50)) illustrating the position of the Fc glycans. Images were rendered in PyMOL version 2.4.

Protein engineering has also been used and includes insertion of reactive glutamine(s) via mutagenesis or incorporation of peptide tags (containing a reactive glutamine) onto the terminal ends of the antibody.^32−34^ These genetic engineering approaches have various shortcomings such as laborious optimization, low yield, provide only a linear architecture, and do not retain the native primary sequence. Consequently, not all antibodies are amenable to the genetic route.

The complete deglycosylation of the N-glycan on Asn297, e.g., by PNGase F, has several disadvantages. First, deglycosylation transforms asparagine (N297) into aspartic acid (D297). This deamidation process results in a negatively charged group at physiological conditions (Scheme 1). The charge variant may lead to numerous issues such as significant structural changes, functional perturbation and immunogenicity, which ultimately may affect the function and efficacy of the antibody conjugates.^12,13,17,20,35−43^

Herein, we present a method that overcomes these drawbacks as well as enables parallel glycan remodeling and dual conjugation. As depicted in Schemes 1 and 2, first, commercial endoglycosidase (EndoS2, EC 3.2.1.96) trims the N-glycan to its core glycan N-acetylglucosamine (GlcNAc). EndoS2 are a family of hydrolases that selectively hydrolyzes N-linked glycans in the Fc region of a native antibody and leaves the innermost GlcNAc intact.^44,45^ Unlike amidase or glycosidase (PNGase F), EndoS2-mediated hydrolysis maintains the neutral asparagine residue (N297). This enzyme is commercially available and applicable to a range of antibody isotypes (e.g., human IgG 1, 2, 3 and 4,^46^ and even goat polyclonal antibodies, unpublished work that is under review), and thus has found practical utility in simplifying analysis,^47^ bioconjugation,^43^ diagnosis^43^ and increased specificity in imaging.^48^ In the second and final steps, mTGase is used to introduce a desired amine-containing group (Scheme 1). mTGase is also commercially available and applicable to a wider range of antibody isotypes (e.g., human IgG 1, 2, 3 and 4; murine IgG 1 and 3, and rat IgG1, 2a, 2b and 2c,^31^ also see Figure S1).

## Results and Discussion

**Figure 1:**
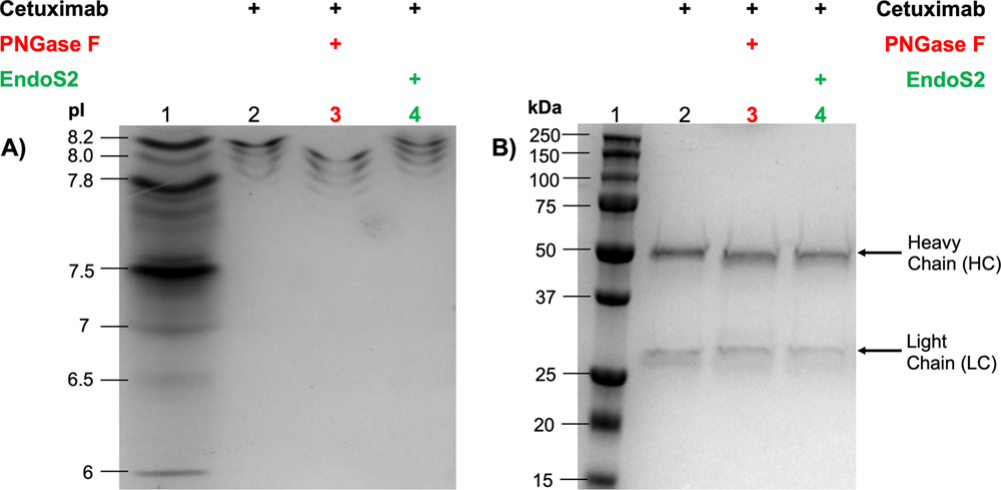
Comparison of PNGase F and EndoS2 mediated hydrolysis of cetuximab. An isoelectric focusing (IEF) gel illustrated a shift in the overall pI of the antibody (i.e., charge profile) after PNGase F hydrolysis (lane 3), whereas EndoS2 hydrolysis (lane 4) of the antibody maintained an indistinguishable pI or charge variant profile from that of its native counterpart (lane 2).

We initially examined the trimming of N-glycans using cetuximab (Erbitux, chimeric IgG1) as a model system. Both endoglycosidase (EndoS2) and amidase (PNGase F) hydrolyze N-glycans on the constant domain (Fc) of cetuximab and various antibodies (IgG1–4), as illustrated by our group and others,^9,30,45^ albeit with several differences. First, hydrolysis or trimming by EndoS2 (an endoglycosidase) retains the innermost glycan N-acetylglucosamine (GlcNAc), whereas hydrolysis by PNGase F removes the glycan completely. Second, hydrolysis by EndoS2 retains the antibody’s asparagine residue (N297), whereas hydrolysis by PNGase F (an amidase) undergoes deamidation which leads to conversion of asparagine residue (N297, with an amide side-chain) into aspartic acid (D297, with a carboxylic acid side-chain). As depicted in Figure 1, we confirmed these processes using isoelectric focusing (IEF). The isoelectric points (pI) of the EndoS2-treated antibody (pI 7.8–8.2; Figure 1, lane 4) were indistinguishable from the unmodified antibody (pI 7.8–8.2; Figure 1, lane 2) and lower (more acidic) for the PNGase F treated antibody (pI 7.6–8.0; Figure 1, lane 3). The pI difference is consistent with the deamidation mediated by PNGase F, resulting in an ∼0.2 difference shift in the pI. It is worth noting that the multiple bands for the unmodified antibody (pI 7.8–8.2; Figure 1, lane 2) are a result of intrinsic charge heterogeneity. Well documented, these variants are a result of various post translational modifications (PTMs) such as deamidation, C-terminal lysine, glycosylation, or other reactive metabolites, as reported by our group and others.^9,13−19,51,52^ Furthermore, EndoS2 mediated reactions had near quantitative conversions, as observed by clean downward shifts in the electrophoretic mobility of the heavy chain (HC) of the antibody using SDS-PAGE (Figure 1B, lane 3, and Figure 3A, lane 2). It is notable that many antibodies (e.g., human IgG 1, 2, 3 and 4) are amenable to hydrolysis or trimming of the N-glycan catalyzed by endoglycosidase.^46^

**Figure 2:**
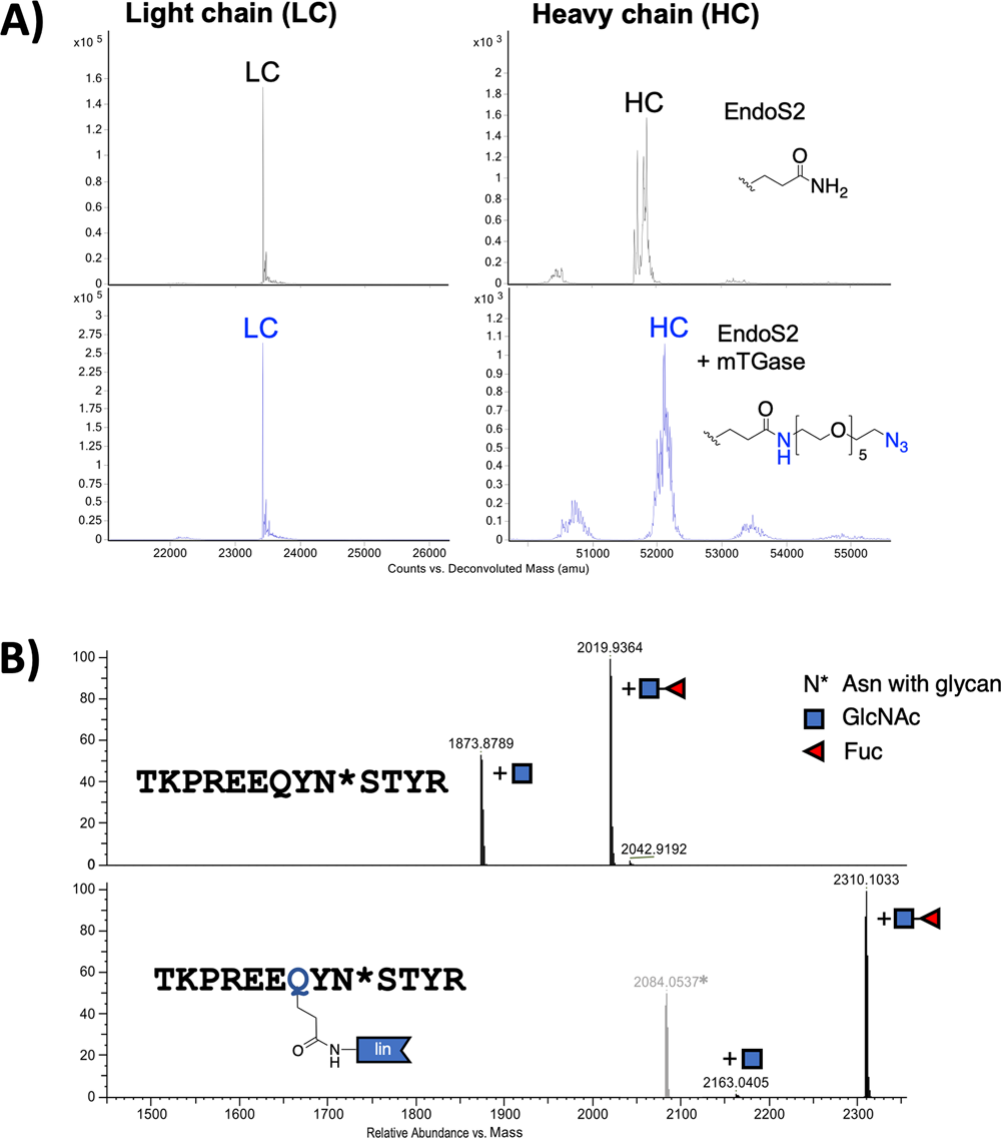
Site-specific transglutaminase-mediated conjugation of a clickable handle (azide) onto cetuximab. (A) Reduced intact mass spectra showed near quantitative installation of an azido group (compound **9**, Figure 1) onto the antibody. (B) Mass spectra of tryptic peptides of EndoS2 treated (top; theoretical monoisotopic mass 1873.8806 Da, observed 1873.8789 Da, difference 0.0017 Da, and 0.9 ppm; theoretical monoisotopic mass 2019.9385 Da, observed mass 2019.9364 Da, difference 0.0021 Da, and 1 ppm) and azido modified Cetuximab by mTGase (bottom; theoretical monoisotopic mass 2163.0449 Da, observed mass 2163.0405 Da, difference 0.0044 Da, and 2 ppm; theoretical monoisotopic mass 2309.1028 Da, observed mass 2309.1011 Da, difference 0.0017 Da, and 0.7 ppm). The asterisk (*) denotes a coeluted unmodified peptide (^189^HKVYACEVTHQGLSSPVTK^207^ in HC; theoretical monoisotopic mass 2084.0593 Da, observed mass 2084.0537 Da, difference 0.0056 Da, and 3 ppm).

**Figure 3:**
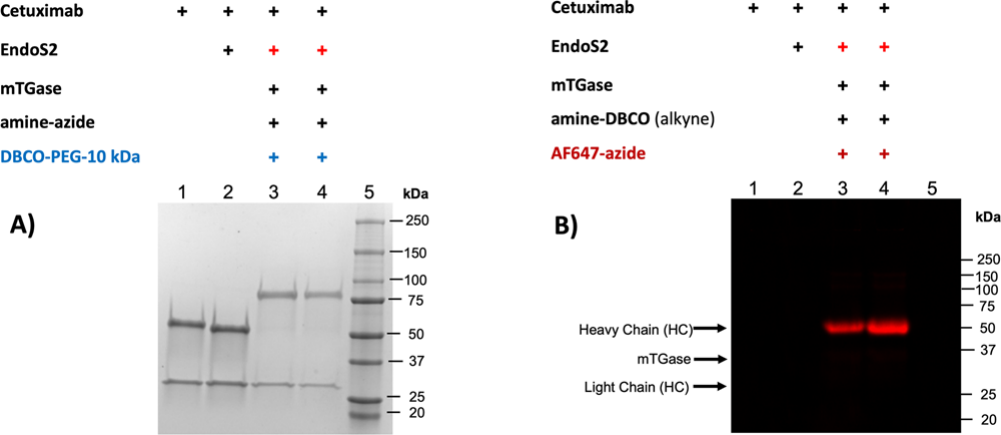
Site-specific transglutaminase-mediated conjugation of polyethylene glycol (PEG) and chromophore (fluorophore) onto cetuximab. (a) SDS-PAGE analysis showed near quantitative installation of a 10 kDa PEG groups onto the antibody (lanes 3 and 4). (b) SDS-PAGE analysis of antibody-fluorophore conjugates showed modification primarily at only the heavy chain (HC) of the antibody; i.e., no modification of the light chain (LC) was detected.

**Scheme 3:**
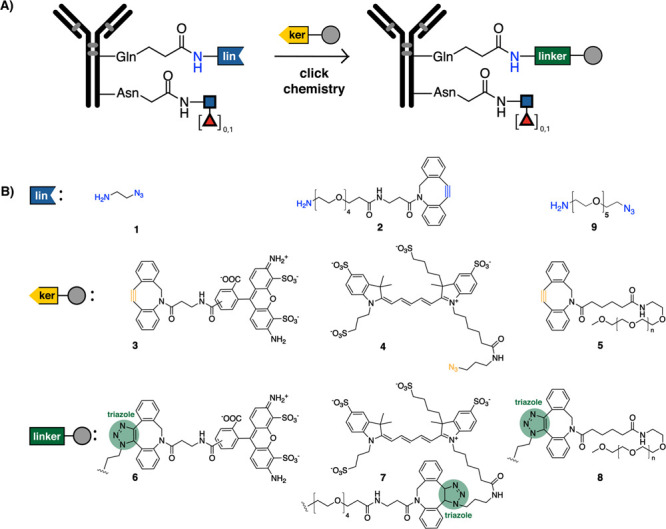
(A) Antibodies Conjugated with an Amine-Containing Clickable Handles (See Scheme 1) Are Further Derivatized via Click Chemistry. (B) Clickable Handles for Assembly of the Antibody Conjugates via Strain-Promoted Azide–Alkyne Cycloaddition (SPAAC) Click Chemistry The trimmed antibody is conjugated to install an amine-containing azide (compounds 1 and 9) or cyclooctyne (compound 2) onto the unsubstituted amide of glutamine (Q295) by mTGase. Modified antibody intermediate is further derivatized to a chromophore with a complementary clickable handle – AF488 (compound 3), AF647 (compound 4) or DBCO-PEG-10 kDa (compound 5). The cyclooctyne and azide form a triazole linker (compounds 6, 7 and 8) in the final conjugates.

Our group, Keillor and others^9,26,53^ have shown that mTGase has broad specificity toward the amines with two main features: primary amines are generally accepted and substituents at the α position significantly slow the reaction down. All amines evaluated were excellent substrates for mTGase (compounds 1, 2 and 9; Scheme 3) after EndoS2 mediated trimming of glycan in cetuximab. These findings suggest that mTGase can readily access the glutamine residue (Q295) after trimming of the innermost glycan. These modifications were confirmed by attachment of a 10 kDa polyethylene glycol (PEG-10 kDa, compound 4) and various fluorophores (compounds 3 and 5) onto cetuximab (Scheme 3) via a two-step click chemistry reaction, namely strain-promoted azide–alkyne cycloaddition (SPAAC).^54−57^ Other click reactions can be utilized in a similar fashion such as tetrazine ligation or copper-catalyzed azide–alkyne cycloaddition (CuAAC).^54−57^

**Figure 4:**
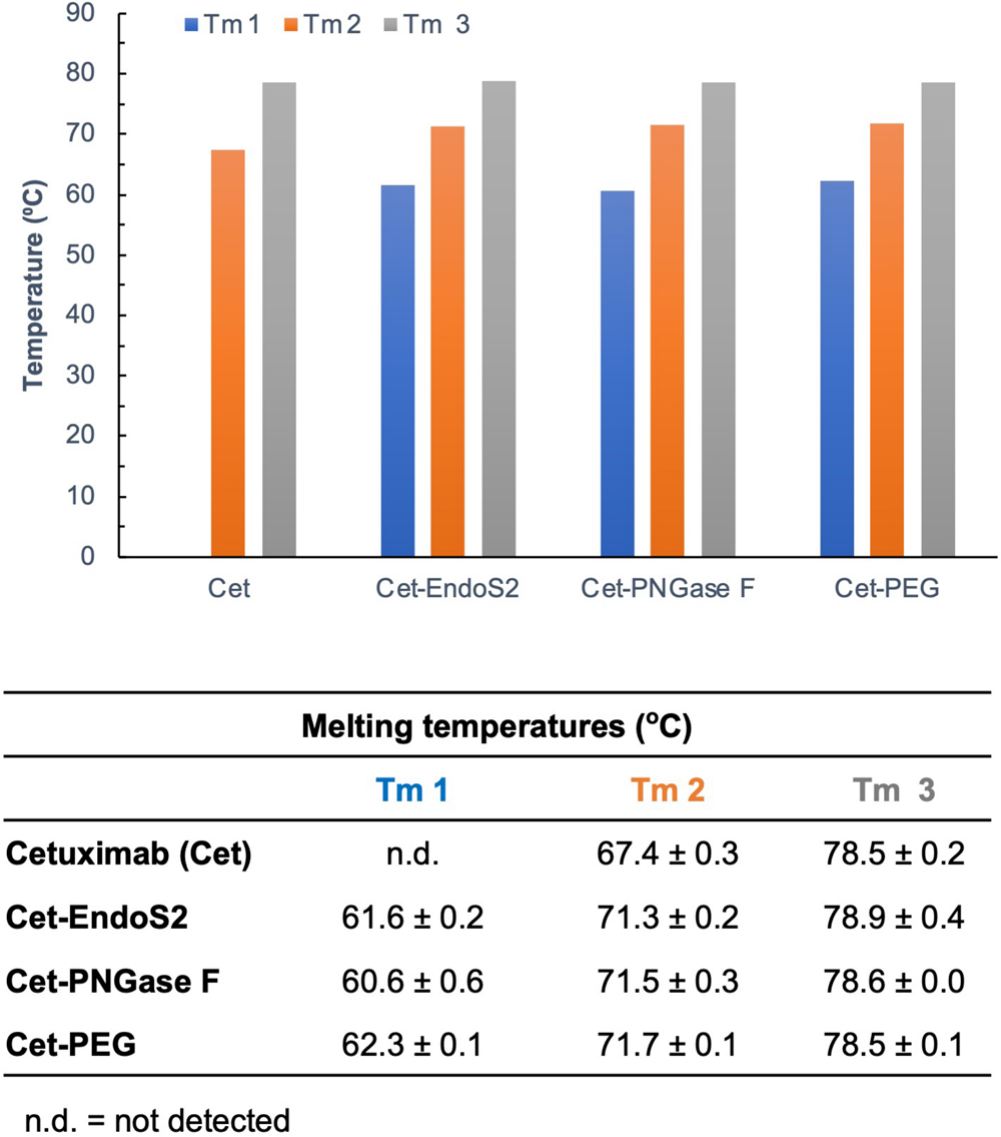
Melting temperatures (*T*_m_) were similar between glycosylated cetuximab (Cet), cetuximab treated with EndoS2 (Cet-EndoS2), cetuximab treated with PNGase F (Cet-PNGase F), and cetuximab incubated with mTGase, 2-azidoethanamine, and then conjugated to a 10 kDa DBCO-PEG-10 kDa (Cet-PEG), as assessed by differential scanning fluorimetry (DSF).^58^ (see Figure S.10).

The mTGase-catalyzed transformation resulted in a modification at a single conserved site (Q295) in the heavy chain (HC) of the antibody (Figures 2 and 3B, lanes 3 and 4; see also Figures S.3, S.4, and S.8.), the same as reported by our group and others.^9,30,31^ For instance, glutamine 295 in cetuximab was quantitatively modified by mTGase and a clickable handle (namely, azido-PEG_5_-amine; compound 9, Scheme 3), as observed by a complete mass shift of the heavy chain (Figure 2A). Whereas the light chain remained unmodified (Figure 2A). Consistently, the two-step click chemistry reaction was also quantitative by a complete shift in the electrophoretic mobility of the antibody’s heavy chain (HC) conjugated to a 10 kDa PEG (Figure 3A, lanes 3 and 4; see also Figure S.2). In other words, the unmodified heavy chain (HC) of the antibody was not observed (Figure 3A, lanes 3 and 4; see also Figure S.2). Both data suggest a drug-to-antibody ratio (DAR) of ∼2. Moreover, cetuximab also contains complex biantennary Fc N-glycans that are highly heterogeneous (both fucosylated and nonfucosylated exist^59^) and most species are fucosylated (Figure 2B). We observed that both fucosylated and nonfucosylated or afucosylated glycan species were modified (Figure 2B), which further expands the utility of our approach. To further examine the scope of our method, infliximab (Remicade, IgG1) was tested and showed similar results (see Figures S.5 and S.7.), as expected from the near identical structures in the Fc regions for these antibodies. Lastly, using differential scanning fluorimetry (DSF),^58^ we observed similar melting temperatures (*T*_m_) between native, endoS2 treated and PEGylated cetuximab (Figure 4 and Figure S.10). This data highlight that our process results in minimal structural alteration of the antibody and thus preservation of the antibody’s function.

**Figure 5:**
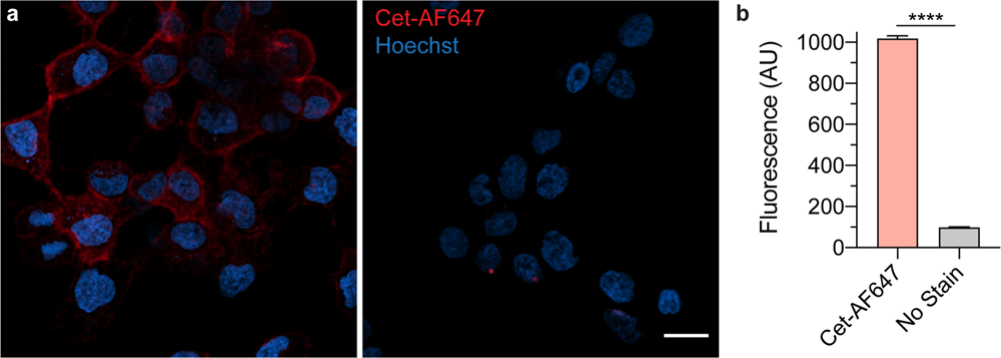
Antibody conjugate’s biological activity is preserved. (A) Ovarian cancer cells (Ovcar3) were stained with cetuximab fluorophore conjugates (Cet-AF647, left) or without (right) and imaged via confocal microscopy. Both groups were stained with Hoechst 33342 nuclear stain. Fluorescence is apparent in the plasma membrane and cytoplasm indicating that Cet-AF647 bound and were internalized. Scale bar, 20 μm. (B) Ovcar3 cells stained with Cet-AF647 and analyzed with flow cytometry are 10-fold brighter than unstained cells (Unpaired, two-tailed *t* test, ****P < 0.0001). Results are mean (±S.D.) fluorescence intensity (*n* = 6 replicates).

The biological function and activity of the antibody conjugates, i.e., cetuximab conjugated to Alexa Fluor 647 (Figure 5b), was assessed via confocal fluorescence microscopy in a cancer cell line (NIH:Ovcar3) that highly expresses epidermal growth factor receptor (EGFR). Under physiological conditions, the antibody conjugates bound and were internalized into the cytoplasm of Ovcar3, as evident from the fluorescence signal that was consistent with our previous PNGase F treated conjugate^9^ (Figure 5; see also Figure S.12). These data confirmed that the antibody conjugate’s biological function was preserved.

## Conclusion

From a method development perspective, the new method presented herein has several noteworthy attributes. First and foremost, prior to this work, it was uncertain whether the trimmed glycan would allow mTGase to access Q295, and, if so, how efficient the transamidation reaction would be. As such, we are pleased to see that near quantitative conversions were observed for both the EndoS2-mediated N-glycan trimming and the mTGase transamidation reactions for multiple antibodies. Second, our results suggest that other glycan trimming enzymes,^43^ may be equally successful. Third, further conjugation via the remaining N-acetylglucosamine (GlcNAc) may be feasible. For example, other saccharrides (with or without bioconjugation handles) can be installed, as demonstrated by GlyClick from Genovis, Site Click from ThermoFisher and GlycoConnect from Lonza (formerly Synaffix).^60,61^ As a result, multiple modification sites and chemistries become available, diversifying the bioconjugation space. Fourth, both EndoS or EndoS2 and mTGase are commercially available and, in combination, applicable to a wide range of antibody isotypes including human IgGs 1–4, mouse IgGs 1 and 3, rabbit IgG, rat IgGs 1 and 2a, bovine IgGs 1 and 2, feline and canine IgGs, as well as equine IgGs 1–7 (see Figure S.1). Last but not least, the glycan remodeling process enables fine-tuning of N-glycans and thus facile modulation of Fc-related biological activities, e.g., antibody dependent cellular cytotoxicity (ADCC) that are mediated by Fc receptors.^62^ Both model antibodies utilized–cetuximab and infliximab–have effector function.^63,64^ To completely abolish this activity, EndoS or EndoS2 can be utilized as reported before.^43^ The abolishment of the effector function results in better imaging agents by decreasing nonspecific binding; for example, reducing binding of the Fc domain to Fcγ receptors on immune cells.^48^ Conversely, to enhance this activity, unnatural glycan substrates (such as oxazolines) can be reintroduced using a glycosynthase and/or removal of fucose using fucosidases to recover the effector function.^65,66^

## Supporting Information Available

Experimental procedures; Figure S.1., Sequence alignment; Figures S.2–S.9, SDS-PAGE; Figure S.10, Thermal shift stability assay; Figures S.11, site of modification by mass spectrometry; Figure S.12, Flow Cytometry (PDF)
