# Supplementary material for: Site-Specific Conjugation of Native Antibody: Transglutaminase-Mediated Modification of a Conserved Glutamine While Maintaining the Primary Sequence and Core Fc Glycan via Trimming with an Endoglycosidase
Source: Bioconjug Chem. 2024 Mar 18;35(4):465–71. doi: 10.1021/acs.bioconjchem.4c00013 (PMC11036358; doi:10.1021/acs.bioconjchem.4c00013)
Supplement: Supplementary file 1 — bc4c00013_si_001.pdf [file bc4c00013_si_001.pdf]

Supporting Information

Site-specific Conjugation of Native Antibody: Transglutaminase-Mediated Modification of a Conserved Glutamine While Maintaining the Primary Sequence and Core Fc Glycan via Trimming with an Endoglycosidase

Amissi Sadiki[†,*], Shanshan Liu[†], Shefali R. Vaidya[†], Eric M. Kercher[‡], Ryan T. Lang[‡], James McIsaac[‡], Bryan Q. Spring[‡], Jared R. Auclair[†], and Zhaohui Sunny Zhou[†,*]

[†]Department of Chemistry and Chemical Biology, Barnett Institute of Chemical and Biological Analysis, Northeastern University, Boston, Massachusetts, USA 02115.

[‡]Translational Biophotonics Cluster, Department of Physics, Department of Bioengineering, Northeastern University, Boston, Massachusetts, USA 02115.

*Correspondence should be sent to sadiki.a@northeastern.edu, z.zhou@northeastern.edu

Table of Contents

1	Materials and methods	4
1.1	Trimming via hydrolysis of N-glycan using endoglycosidase S2 (EndoS2)	4
1.2	Transglutaminase-mediated transamidation	4
1.3	Strain-promoted azide-alkyne cycloaddition (SPAAC) click reaction	5
1.4	Characterization of the conjugates by SDS-PAGE	5
1.5	Characterization of the conjugates by IEF	6
1.6	Characterization of the conjugates by a thermal shift stability assay	7
1.7.	Characterization of the conjugates by mass spectrometry	7
2.	Cancer cell based activity assay	19
2.1.	Antibody conjugates activity via flow cytometry	19
2.2.	Antibody conjugates internalization assessment via confocal microscopy	19
3.	References	22

human IgG 1	PREEQYNST
human IgG 2-4	PREEQFNST
mouse IgG 1	PREEQFNST
mouse IgG 3	PREAQYNST
rabbit IgG	LREQQFNST
rat IgG 1	PREEQYNST
rat IgG 2a	APEKQSNST
bovine IgG 1-2	PREEQFNST
feline IgG	PREEQFNST
canine IgG	SREQQFNGT
equine IgG 1	PKEEQFNST
equine IgG 2	QREAQFNST
equine IgG 3	PNEEQNNST
equine IgG 4	PKQEQFNST
equine IgG 5	PKEEQFNST
equine IgG 6	AKEKQDNST
equine IgG 7	PKQEQNNST

Figure S.1. Amino acid sequence alignment of human, mouse, rabbit, and equine IgG's Fc fragments [1,2]. Sites of glutamine modification mediated by transglutaminase (Gln, Q; Q295 for IgG1) are highlighted in red and the N-glycosylation sites (Asn, N; N297 for IgG1) are highlighted in green. The sequences were aligned using EMBL-EBI Clustal Omega [3].

1. Materials and Methods

The concentrations of the peptides and proteins were determined using UV absorption at 280 nm and extinction coefficients based on amino acid sequences. All aqueous solutions were prepared using Milli-Q water. All cell culture methods were performed with aseptic technique in a biosafety cabinet.

1.1 Trimming or hydrolysis of N-glycan using Endoglycosidase S2 (EndoS2)

The reaction contained 18 μM cetuximab (Erbitux, Selleckchem, A2000) or 17 μM infliximab (Remicade, European Pharmacopoeia, Y0002047) and 25 mM Tris-buffered saline (TBS) pH 7.4, and was initiated with immobilized EndoS2 GlycINATOR (E.C. 3.2.1.96, Genovis; A0-GL6-010, per manufacturer instructions) at 37 °C for 3 h and 40 min. To remove excess unreacted reagents, each reaction mixture was desalted using 30 kDa molecular weight cut-off (MWCO) centrifugal filters (Amicon unit, UFC503096) into 25 mM TBS pH 7.4, prior to the transamidation reaction.

1.2 Transglutaminase-mediated Transamidation

The transamidation reaction contained 25 mM TBS pH 7.4 or 100 mM Tris pH 8, 100 mM 2-azidoethanamine (compound **1**, Scheme 3; Acrotein ChemBio, AS00696) or 5 mM dibenzylcyclooctyne-PEG_4-amine (compound **2**, Scheme 3; Click Chemistry Tools, A103P) or 10 mM Azido-PEG_5-amine (compound **9**, Scheme 3; Broad Pharm BP-20590), and 1.6 μM

trimmed cetuximab or 1.5 μM trimmed infliximab, and was initiated with 1.7 μM microbial transglutaminase (mTGase, EC 2.3.2.13, Uniprot P81453; Ajinomoto, ACTIVA-TI formulation or Zedira, T255) and incubated at 37 °C for 12.5–24 h. To remove excess unreacted reagents, each reaction mixture was desalted using 50 kDa MWCO centrifugal filters (Amicon unit, UFC505024) into 25 mM TBS pH 7.4 or 100 mM Tris pH 8, prior to the click reaction.

1.3 Strain-Promoted Azide-Alkyne cycloaddition (SPAAC) Click Reaction

The reaction contained 25 mM TBS pH 7.4, 200 μM of Alexa Fluor 647 ® azide (AF647, compound **3**, Scheme 3.3.1., Click Chemistry Tools, 1299) or Alexa Fluor 488 ® dibenzylcyclooctyne (AF488, compound **4**, Scheme 3.3.1., Genovis, L1-F01-025, per manufacturer instructions) or 10 kDa polyethylene glycol (PEG) dibenzylcyclooctyne (DBCO-PEG-10 kDa, compound **5**, Scheme 3.3.1., Click Chemistry Tools, A119), and 1.5 μM cetuximab or infliximab modified by mTGase and incubated at 22.5 °C for 16–18 h. To remove excess unreacted reagents, each reaction mixture was desalted using 50 kDa MWCO centrifugal filters into 25 mM TBS pH 7.4.

1.4 Characterization of the Conjugates by SDS-PAGE

Sodium dodecyl sulfate–polyacrylamide gel electrophoresis (SDS-PAGE) was performed using a Bio-Rad Mini-PROTEAN 3 system or Criterion Cell. First, the reaction mixture was incubated with SDS Sample Buffer at 80 °C for 10 min. For reducing and nonreducing gels, 4X

reducing SDS sample buffer (Boston Bio Products, BP-110R) and 2X nonreducing SDS sample buffer (Bio- Rad, 1610737) were used, respectively. Second, the samples were loaded into 12% Tris-tricine precast protein gels (Bio-Rad, 4561044) or 4–15% Tris-glycine precast protein gels (Biorad, 5671083). Precision Plus Protein™ Dual Xtra Prestained Protein Standards (Bio-Rad, 1610377) were used for mass calibration. Electrophoresis was then performed at 200 V for 20 min. The gel was stained by Coomassie R250 and then destained using 10% acetic acid and 40% methanol. The gels were imaged using an iBright FL1000 Imaging system (Thermo Fisher Scientific).

1.5 Characterization of the Conjugates by IEF

Isoelectric focusing (IEF) was performed using a Bio-Rad Criterion system. First, the reaction mixture was mixed in 1:1 dilution with isoelectric focusing (IEF) sample buffer (Bio-Rad, 1610763). Second, the samples were loaded into pH 3–10 Criterion IEF precast gel and placed into the cell. Then, 1X Anode buffer (Bio-Rad, 1610761) and 1X Cathode buffer (Bio-Rad, 1610762) were placed in the upper and lower chamber of the criterion cell, respectively. Third, IEF was run initially at 100 V for 1 h to initiate desalting of the sample, followed by higher voltage at 250 V for 1 h to mobilize the antibody, and lastly 500V for 30 min to complete electro-focusing. The gel was stained by Coomassie R250 and Crocein Scarlet and then destained using 10% acetic acid and 40% methanol. The gels were imaged using an iBright FL1000 Imaging system.

1.6. Characterization of the Conjugates by Thermal Shift Assay

All thermal stability assays were carried out using a 96-well plate with a final volume of 20 µL on a Bio-Rad CFX96 Real-Time PCR Detection System. Samples for all antibody conjugates were prepared at concentrations of 0.2 mg/mL. The assay was performed in PBS pH 7.4 with a 10x concentration of SYPRO Orange (Invitrogen).[4] Samples were heated from 20 to 100 ^{0}C at a rate of 0.5 ^{0}C/min with fluorescence measurements recorded at 0.2-degree increments. Melting temperatures were calculated from $\Delta F/\Delta T$ values based on a minimum of three replicates. Standard deviation is reported with listed values.

1.7. Characterization of the conjugates by mass spectrometry

1.7.1. Intact protein analysis: Mass spectrometry was performed using an Agilent 1280 liquid chromatography system coupled with an Agilent 6560 QToF mass spectrometer. The EndoS treated cetuximab and its conjugates were first reduced by incubating with 10 mM dithiothreitol (DTT) at 56 ^{o}C for 30 min. The mixtures were desalted using a ZipTip C4 desalting column, then reconstituted in water with 10% acetonitrile and 0.1% formic acid. 5-10 µL of samples were injected into the LC/MS system at 0.3 mL/min flow rate. The chromatographic separation was run under a 20-minute gradient from 10 to 80% acetonitrile in water with 0.1% formic acid, the samples eluted from LC were introduced via an electrospray ion source to the QToF for mass spec acquisition. The intact protein data was analyzed using Agilent MassHunter BioConfirm software 10.

1.7.2. Peptide mapping analysis: The antibody samples were reduced and denatured by incubating the antibody and its conjugates with 2 M urea and 10 mM DTT for 1 hour at 56 ºC. The mixtures were then diluted 4x with 50 mM ammonium bicarbonate to make the final concentration of urea at 0.5 M. Trypsin was added to the mixture at an enzyme: protein ratio of 1:50 (w/w), the digestion was conducted at 37 °C for 15 hours. The resulting peptide mixture was desalted with Thermo Pierce™ C18 spin columns, and then reconstituted in water with 10% acetonitrile and 0.1% formic acid prior to mass spec acquisition. 10 µL of samples was loaded onto a C18 UPLC column and separated via a 45-min gradient of Acetonitrile/water with 0.1% of formic acid. Peptide mapping was acquired using a Thermo Orbitrap Exploris 120 mass spectrometry system. Data analysis was performed using Thermo BioPharma Finder software 5.2. All reagents used during the mass spectrometry analysis were LC/MS level.

	reduced					non-reduced				reduced		
**Lanes**	**1**	**2**	**3**	**4**	**5**	**6**	**7**	**8**	**9**	**10**	**11**	**12**
**Cetuximab**	+	+	+	+	ladder	+	+	+	+		+	+
**EndoS2**		+	+	+			+	+	+			+
**mTGase**			+	+				+	+	+		
**amine-azide**			+	+				+	+			
**DBCO-PEG-10 kDa**			+	+				+	+			

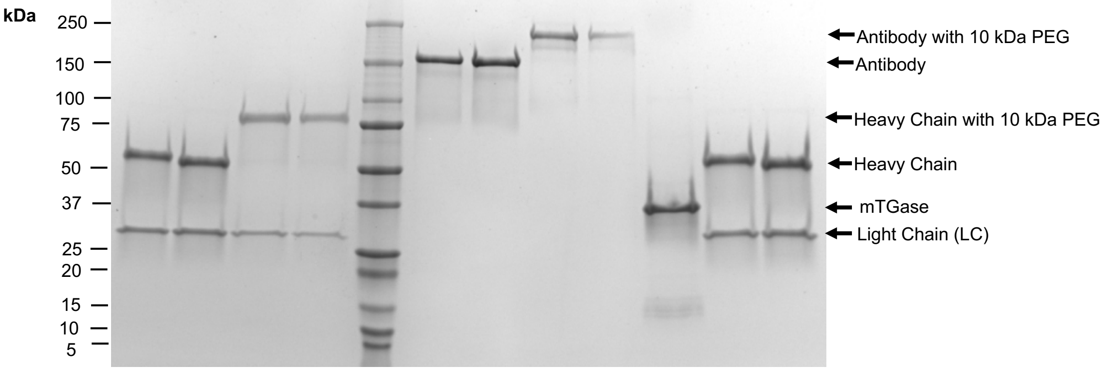

Figure S.2. Reducing SDS-PAGE gel image of cetuximab-PEGylated conjugates with Coomassie staining. Lanes: 1) cetuximab, 2) cetuximab treated with endoglycosidase S2 (EndoS2), 3) and 4) trimmed cetuximab incubated with microbial transglutaminase (mTGase), 2-azidoethanamine, and then 10 kDa polyethylene glycol (PEG) dibenzylcyclooctyne (DBCO-PEG-10 kDa), 5) Molecular weight standards; non-reduced, 6) cetuximab, 7) cetuximab treated with endoglycosidase S2 (EndoS2), 8) trimmed Cetuximab incubated with transglutaminase (mTGase), 2-azidoethanamine, and then 10 kDa polyethylene glycol (PEG) dibenzylcyclooctyne (DBCO-PEG-10 kDa); reduced, 9) cetuximab, 10) cetuximab treated with endoglycosidase S2 (EndoS2). Based on the shift by PEGylation (lanes 3, 4, 8 and 9), the transglutaminase-mediated and click chemistry reactions yields were quantitative.

Lanes	1	2	3	4	5
**Cetuximab**	+	+	+	+	ladder
**EndoS2**		+	+	+	
**mTGase**			+	+	
**amine-DBCO** (alkyne)			+	+	
**AF647-azide**			+	+	

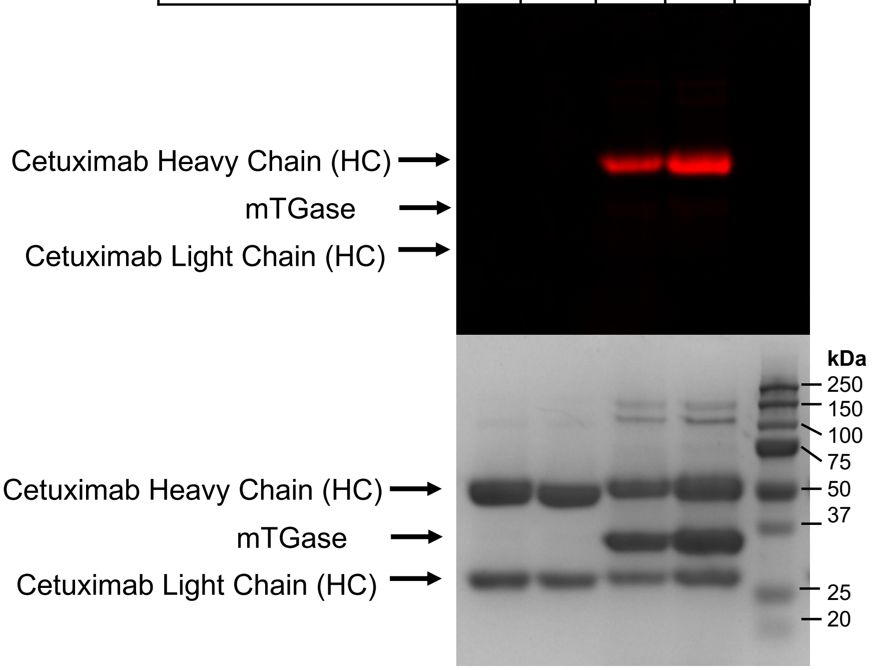

Figure S.3. Reducing SDS-PAGE gel image of cetuximab-chromophore conjugates: (a) top, fluorescence imaging (excitation 608-632 nm and emission 675-720 nm), and (b) bottom, Coomassie staining. Lanes: 1) cetuximab, 2) cetuximab treated with endoglycosidase S2 (EndoS2), 3) and 4) trimmed cetuximab incubated with microbial transglutaminase (mTGase) and dibenzylcyclooctyne-PEG4-amine, and then Alexa Fluor 647 azide, 5) Molecular weight standards.

Lanes	1	2	3	4	5
Cetuximab	ladder	+	+	+	
EndoS2			+	+	
mTGase				+	+
amine-DBCO				+	
azide-AF647				+	

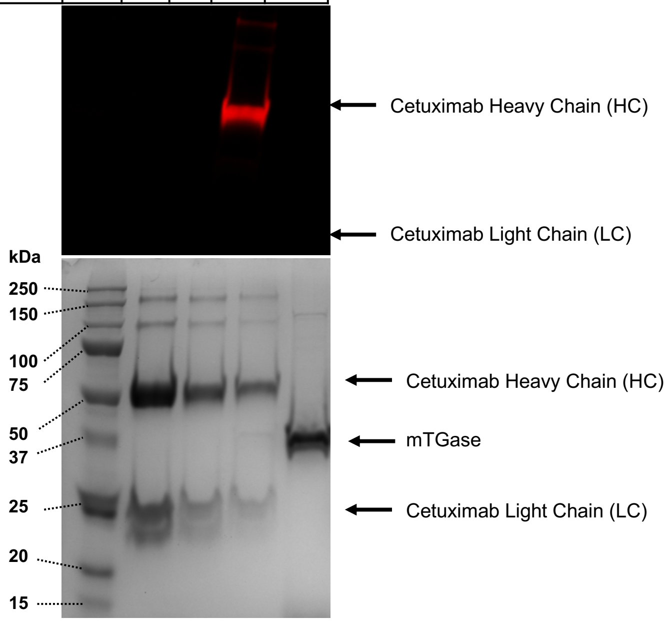

Figure S.4. Reducing SDS-PAGE gel image of cetuximab-chromophore conjugates: (a) top, fluorescence imaging (excitation 608-632 nm and emission 675-720 nm), and (b) bottom, Coomassie staining. Lanes: 1) Molecular weight standards, 2) cetuximab, 3) cetuximab treated with endoglycosidase S2 (EndoS2), 4) trimmed Cetuximab incubated with microbial transglutaminase (mTGase) and dibenzylcyclooctyne-PEG4-amine, and then Alexa Fluor 647 azide, 5) mTGase.

Lanes	1	2	3	4
Infliximab	ladder	+	+	+
EndoS2				+
mTGase				+
amine-DBCO				+
azide-AF647				+

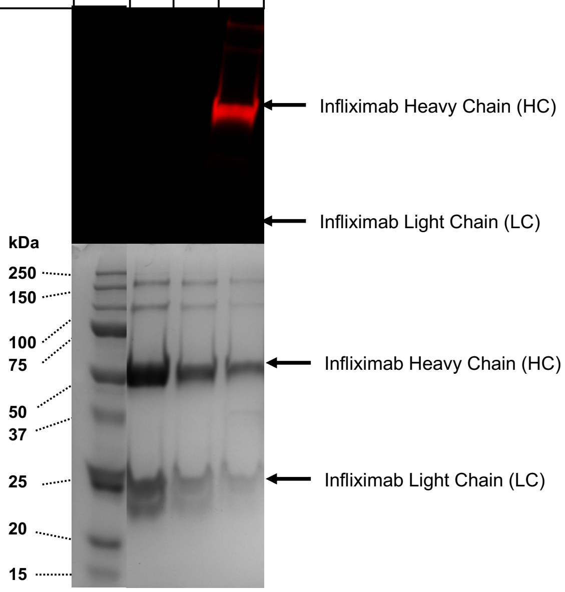

Figure S.5. Reducing SDS-PAGE gel image of infliximab-chromophore conjugates: (a) top, fluorescence imaging (excitation 608-632 nm and emission 675-720 nm), and (b) bottom, Coomassie staining. Lanes: 1) Molecular weight standards, 2) infliximab, 3) infliximab treated with endoglycosidase S2 (EndoS2), 4) trimmed infliximab incubated with microbial transglutaminase (mTGase) and dibenzylcyclooctyne-PEG4-amine, and then Alexa Fluor 647 azide.

Lanes	1	2	3
Cetuximab	ladder	+	+
EndoS2		+	+
mTGase			+
amine-DBCO			
azide-AF647			+

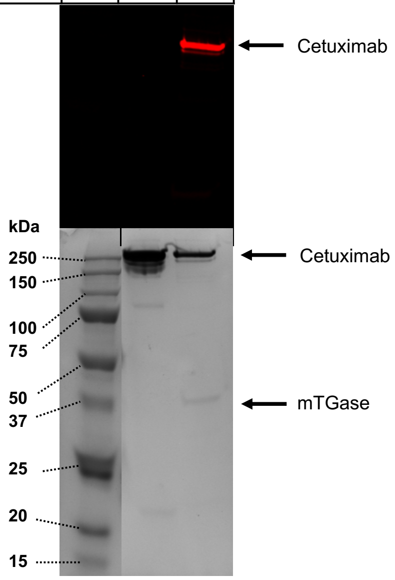

Figure S.6. Nonreducing SDS-PAGE gel image of cetuximab-chromophore conjugates: (a) top, fluorescence imaging (excitation 608-632 nm and emission 675-720 nm), and (b) bottom, Coomassie staining. Lanes: 1) Cetuximab treated with endoglycosidase S2 (EndoS2), 2) trimmed cetuximab incubated with transglutaminase (TGase) and dibenzylcyclooctyne-PEG4-amine, and then Alexa Fluor 647 azide.

Lanes	1	2	3	4
Infliximab		+	+	+
EndoS2			+	+
mTGase				+
amine-azide				+
DBCO-AF488				+

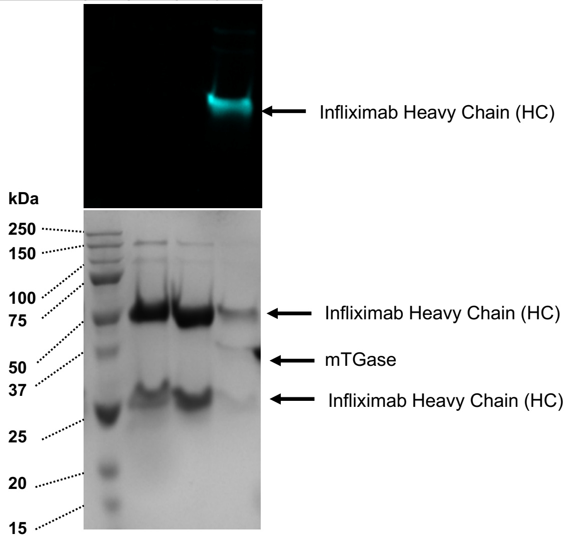

Figure S.7. Reducing SDS-PAGE gel image of infliximab-chromophore conjugates: (a) top, fluorescence imaging (excitation 455-485 nm and emission 508-557 nm), and (b) bottom, Coomassie staining. Lanes: 1) Molecular weight standards, 2) infliximab, 3) infliximab treated with endoglycosidase S2 (EndoS2), 4) trimmed infliximab incubated with microbial transglutaminase (TGase) and 2-azidoethanamine, and then Alexa Fluor 488 dibenzylcyclooctyne.

Lanes	1	2	3	4
Cetuximab	+	+	+	ladder
EndoS2		+	+	
mTGase			+	
amine-azide			+	
DBCO-AF488			+	

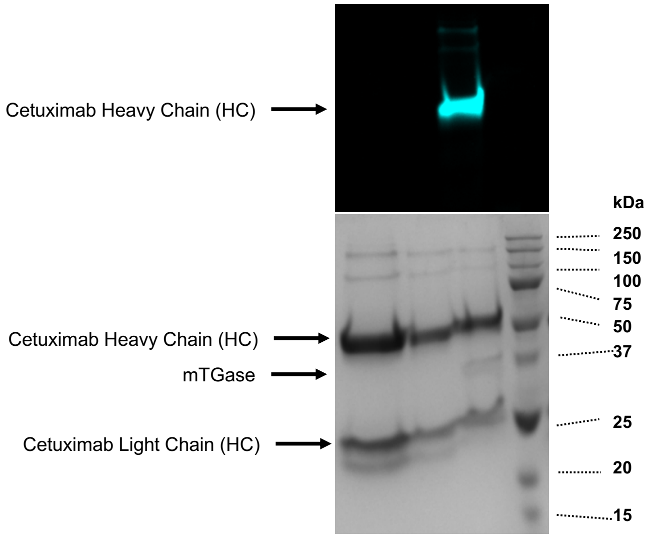

Figure S.8. Reducing SDS-PAGE gel image of cetuximab-chromophore conjugates: (a) top, fluorescence imaging (excitation 455-485 nm and emission 508-557 nm), and (b) bottom, Coomassie staining. Lanes: 1) Cetuximab, 2) cetuximab treated with endoglycosidase S2 (EndoS2), 3) trimmed cetuximab incubated with transglutaminase (TGase) and 2-azidoethanamine, and then Alexa Fluor 488 dibenzylcyclooctyne, 4) Molecular weight standards.

Lanes	1	2	3
Cetuximab	ladder	+	+
EndoS2		+	+
mTGase			+
amine-DBCO			+
azide-AF488			+

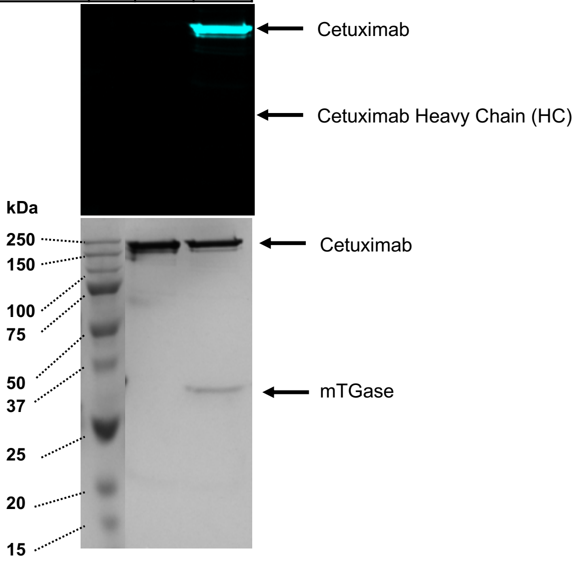

Figure S.9. Nonreducing SDS-PAGE gel image of cetuximab-chromophore conjugates: (a) top, fluorescence imaging (excitation 455-485 nm and emission 508-557 nm), and (b) bottom, Coomassie staining. Lanes: 1) Cetuximab treated with EndoS2, 2) trimmed cetuximab incubated with transglutaminase and 2-azidoethanamine, and then Alexa Fluor 488 dibenzylcyclooctyne.

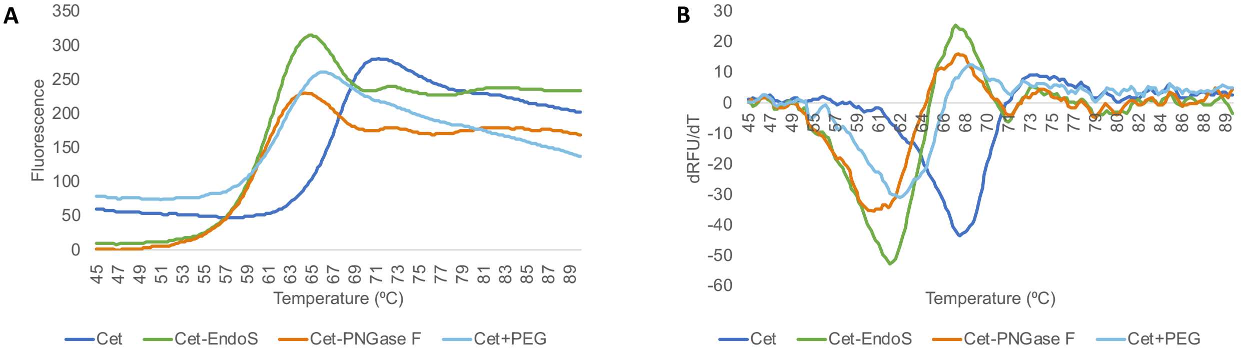

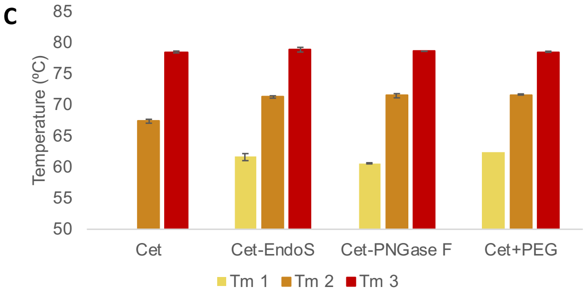

D

	Tm 1 (ºC)	Tm 2 (ºC)	Tm 3 (ºC)
Cet	n.d.	67.37 ± 0.31	78.50 ± 0.15
Cet-EndoS2	61.60 ± 0.16	71.27 ± 0.19	78.87 ± 0.38
Cet-PNGase F	60.60 ± 0.57	71.47 ± 0.34	78.60 ± 0.00
Cet-PEG	62.33 ± 0.09	71.67 ± 0.09	78.53 ± 0.09

n.d. = not detected

Figure S.10. (A) and (B) Thermal shift stability assay of cetuximab (Cet), cetuximab treated with EndoS2 [Cet-EndoS2; also named trimmed cetuximab], cetuximab treated with PNGase F (Cet-PNGase F), and lastly, trimmed cetuximab incubated with mTGase, 2-azidoethanamine, and then 10 kDa DBCO-PEG-10 kDa (Cet-PEG). (C) and (D) Summary of melting temperatures (Tm) obtained from the assay. Melting temperatures were assessed using Sypro Orange dye fluorescence.

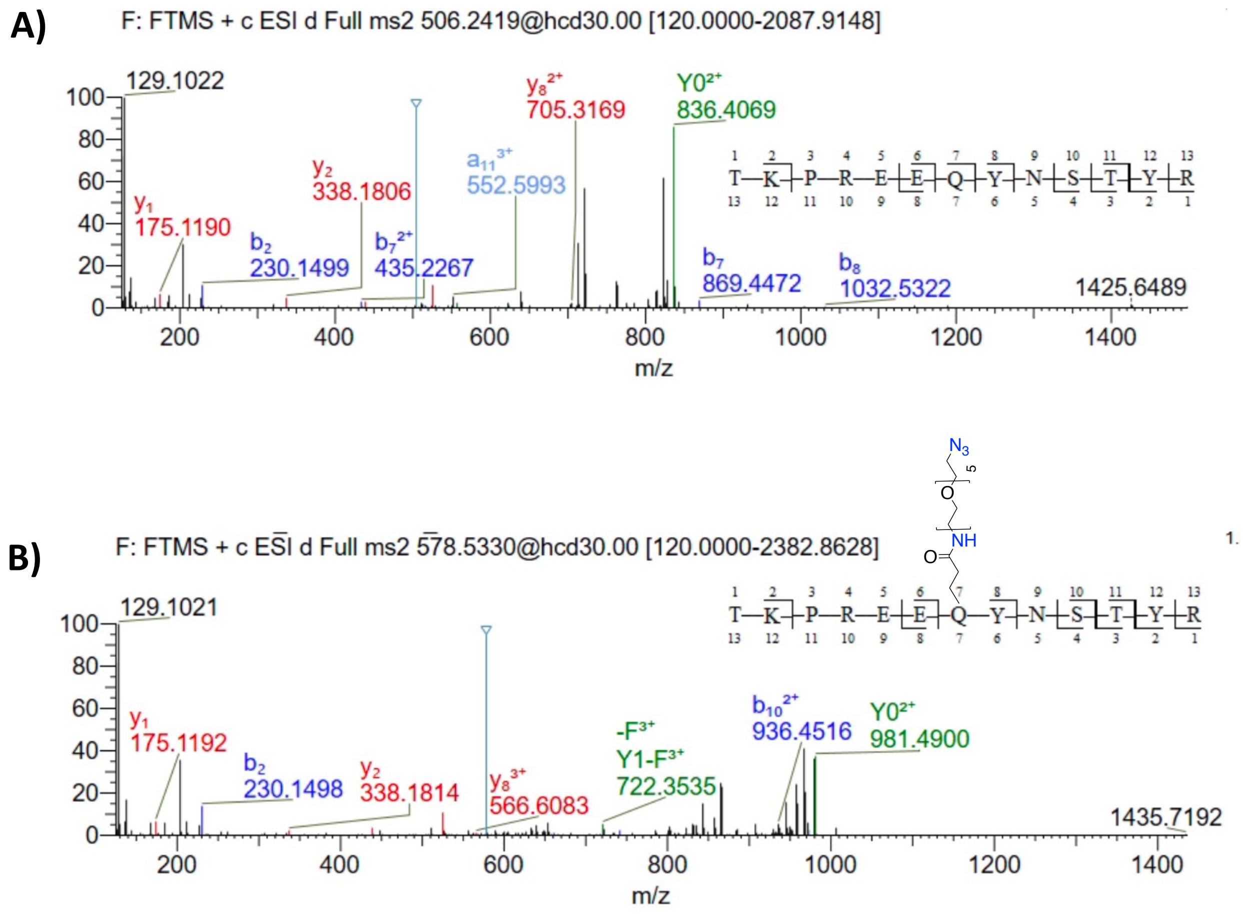

Figure S.11. ESI MS/MS spectra of Q295 and N297 containing peptide (TKPREE**Q**YN*STYR) of (A) cetuximab treated with EndoS2 and (B) trimmed cetuximab incubated with mTGase and then azido-PEG$_5$-amine.

2. Cancer Cell-Based Activity Assay

NIH:OVCAR-3 (Ovcar3) ovarian carcinoma cells (HTB-161, American Type Culture Collection, ATCC) were cultured in the recommended RPMI-1640 media (ATCC, 30-2001) supplemented with 20% heat-inactivated fetal bovine serum (R&D Systems, S11150H) and 0.01 mg/mL bovine insulin (Sigma-Aldrich, I0516) in a humidified incubator at 37 °C and 5% CO_2. Media was replenished every 2–3 days and cells were passaged in T75 culture-treated flasks (Thermo Scientific, 12-565-350) at 70-90% confluency.

2.1. Antibody Conjugates Activity via Flow Cytometry

Ovcar3 cells were harvested and resuspended at 5×10^5 cells per mL in 1 mL of media with and without 0.5 ng/µL of cetuximab-AF647 (Cet-AF647). Cells were incubated for 1 h at 4 °C in the dark. Each sample was washed twice and resuspended in 1 mL of phosphate buffered saline (10-010-023, Gibco). Cells were analyzed via flow cytometer (Attune NxT, Thermo Fisher) equipped with 635 nm laser. Stained and unstained cells were sampled 6 times. Cells were gated and measured for mean AF647 fluorescence (see Figure S.11.) using Attune NxT software. Results were compiled and analyzed in Prism 8 (GraphPad Software).

2.2. Antibody Conjugates Internalization Assessment via Confocal Microscopy

Ovcar3 cells were harvested and plated in a 24-well plate with #1.5 cover glass (P24-1.5H-N, Cellvis) at 30,000 cells per well in 1 mL of cell culture media. Cells were incubated for

48 h. Cet-AF647 staining solution was prepared by diluting Cet-AF647 to 5 ng/μL in cell culture media. Media from each well was aspirated and replaced with 200 μL of 5.0 ng/μL staining solution or with fresh media and incubated for 1 h at 37 °C prior to imaging. 15 min prior to imaging, 2.5 μg/mL of Hoechst 33342 (Invitrogen, H3570) was added to each well. The plate was then imaged using confocal fluorescence microscopy (Zeiss LSM800) with a 40× objective. All imaging parameters were kept consistent throughout imaging.

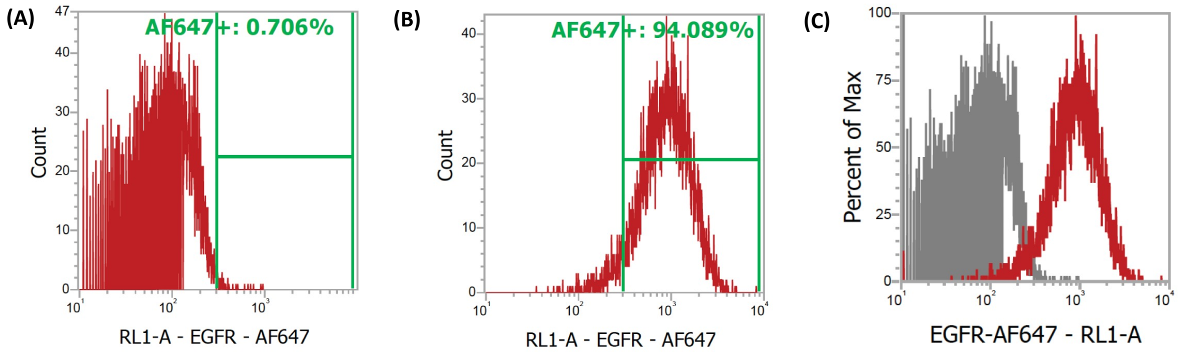

Figure S.12. Analysis of Ovcar3 cells via Flow Cytometry. AF647 fluorescence histograms for (a) unstained cells and (b) cells stained with Cet-AF647. (c) Single exemplary overlays comparing stained (in red) and unstained (in grey) cell populations. Each group was sampled 6 times and mean fluorescence intensity was recorded for further analysis (see manuscript, Figure 5).

3. References

1. Miyakawa, S., Nomura, Y., Sakamoto, T., Yamaguchi, Y., Kato, K. Yamazaki, S., Nakamura, Y. (2008) Structural and molecular basis for hyperspecificity of RNA aptamer to human immunoglobulin G. *RNA* 14, 6, 1154-63.
2. Nagaoka, M., Akaike, T. (2003) Single amino acid substitution in the mouse IgG1 Fc region induces drastic enhancement of the affinity to protein A. *Protein Eng* 16, 4, 243-5.
3. Madeira, F., Mi Park, Y., Lee, J., Buso, N., Gur, T., Madhusoodanan, N., Basutkar, P., Tivey, A. R. N., Potter, S. C., Finn, R. D., Lopez, R. (2019) The EMBL-EBI search and sequence analysis tools APIs in 2019. *Nucleic Acids Res* 47, W636-W641.
4. Ericsson, U. B., Hallberg, M., Detitta, G. T., Dekker, N., Nordlund, P. (2006) Thermofluor-based high-throughput stability optimization of proteins for structural studies. *Anal Biochem* 357, 289.
